# New statistical methods for estimation of recombination fractions in F_2_ population

**DOI:** 10.1186/s12859-017-1804-8

**Published:** 2017-10-03

**Authors:** Yuan-De Tan, Xiang H. F. Zhang, Qianxing Mo

**Affiliations:** 1 Dan L. Ducan Cancer Center, Baylor College of Medicine, Houston, TX USA; 2 Lester and Sue Smith Breast Center, Baylor College of Medicine, Houston, TX USA; 3 Department of Molecular and Cellular Biology, Baylor College of Medicine, Houston, TX USA; 4 McNair Medical Institute, Baylor College of Medicine, Houston, TX USA; 5 Department of Medicine, Baylor College of Medicine, Houston, TX USA

**Keywords:** Dominant marker, Codominant marker, Gamete frequency, EM algorithm, ELS algorithm

## Abstract

**Background:**

Dominant markers in an F_2_ population or a hybrid population have much less linkage information in repulsion phase than in coupling phase. Linkage analysis produces two separate complementary marker linkage maps that have little use in disease association analysis and breeding. There is a need to develop efficient statistical methods and computational algorithms to construct or merge a complete linkage dominant marker maps. The key for doing so is to efficiently estimate recombination fractions between dominant markers in repulsion phases.

**Result:**

We proposed an expectation least square (ELS) algorithm and binomial analysis of three-point gametes (BAT) for estimating gamete frequencies from F_2_ dominant and codominant marker data, respectively. The results obtained from simulated and real genotype datasets showed that the ELS algorithm was able to accurately estimate frequencies of gametes and outperformed the EM algorithm in estimating recombination fractions between dominant loci and recovering true linkage maps of 6 dominant loci in coupling and unknown linkage phases. Our BAT method also had smaller variances in estimation of two-point recombination fractions than the EM algorithm.

**Conclusion:**

ELS is a powerful method for accurate estimation of gamete frequencies in dominant three-locus system in an F_2_ population and BAT is a computationally efficient and fast method for estimating frequencies of three-point codominant gametes.

**Electronic supplementary material:**

The online version of this article (10.1186/s12859-017-1804-8) contains supplementary material, which is available to authorized users.

## Background

A great advance has been made in building genetic maps of various species due to the development of large-scale molecular marker technologies [1–7] and statistical methods [4, 8–18]. However, mapping of numerous molecular markers has been complicated by linkage phases of dominance [14–16, 19]. In two-point analysis, markers in repulsion phase provide quite less linkage information than in coupling phase [14, 15, 20, 21]. This is especially true for dominant markers in F_2_ population [14]. In practical mapping experiments, although the linkage phase for each dominant marker is random, a half of markers are derived from one of two coupling phases. The phase between couplings is repulsion [14, 15]. This situation results in two separate partner linkage maps for dominant markers: high linkage information content of markers in the coupling phase and low linkage information content of markers in the repulsion phase. Thus one has to build two complementary linkage maps [14, 15, 21, 22]. To date, there has not yet been an effective way to integrate both into a complete map. Mester et al. [15] attempted to use pairs of codominant and dominant (CD) markers to merge such two complementary maps because pairs of the CD markers in repulsion phase have much higher linkage information content than pairs of dominant-only markers in repulsion phase. However, this strategy demands that all dominant markers be paired with codominant markers, which is not a general case in mapping practice, otherwise, local and global disturbance will then violently affect the reliability of the integrated map.

The two-point analysis implemented by the expectation maximization (EM) algorithm [11–13, 23–25] is a highly powerful approach to estimate recombination fractions between codominant loci and between dominant loci in coupling phase, but the EM algorithm has very low power in estimation of recombination fractions between dominant loci in repulsion phase. This is because it is difficult for the EM algorithm to distinguish genotypes in coupling phase from those in repulsion phase for dominant markers.

Therefore, the key of developing a powerful method for mapping dominant loci in an intersection population is to overcome the difficulty of distinguishing coupling phase from repulsion phase. Since two-point analysis, as pointed out above, performs very poorly in the estimation of recombination fractions between dominant loci, three-point analysis is alternatively taken into account. However, few three-point EM algorithms can be applied to dominant markers because dominant markers are less informative for maximum likelihood estimation [26]. One effective way to carry out three-point analysis is to dissect three-point genotypes into various gamete components that are informative for distinction between coupling and repulsion phases, and then, to estimate their frequencies. With these estimated gamete frequencies, one can immediately estimate recombination fractions between dominant loci in couple and repulsion phases. A key to this strategy is to obtain estimate of gamete frequencies. On the basis of dissection of genotypes, Tan and Fu proposed a binomial analysis of three-point (BAT) to estimate frequencies of dominant gametes [19]. However, this binomial approach is limited to the frequency of the three-point recessive gamete abc. The accuracy of estimation is completely dependent on the observed frequency of its phenotype (aabbcc). We have developed a new method called “expectation least square” (ELS) to address this problem. ELS estimation, similarly to expectation maximum algorithm, is realized on the basis of Tan and Fu’s BAT method [19]. That is, the expectation of phenotype frequencies can be given by using Eqs. (1-9) in the BAT of Tan and Fu [19], and the difference between estimated and expected values of phenotype frequencies is given using least square. The expectation and least square steps are iterated so that the difference between estimated and expected values is less than tolerant value. In addition, we have also developed a fast binomial approach to estimate frequencies of codominant gametes.

## Methods

### Real data collection

Mouse genotype data: A RFLP dataset of 333 F_2_ mice was obtained from MAPMAKER/EXP (version 3.0b) [13].

### Simulation

For dominant loci, we just took unknown phase into account in simulation and followed a point process model [27] and scheme of Tan and Fu [19] to perform simulations. In *N* F_1_ meioses, recombination events occurred at random between two adjacent loci. Here for the simplicity, we allowed for only independent crossovers during procedure of recombination occurrence between nonsister chromatides. We generated *N* F_2_ individuals with ratio = phenotype A: phenotype a = 3:1 at each dominant locus or A(homozygote): H(heterozygote): B(homozygote) = 1:2:1 at each codominant locus. We set three levels for sample size: *N* = 100, 200, and 300 F_2_ individuals and 100 iterations and used variance (equivalent to mean square error, MSE) that quantifies deviation of estimated recombination fraction between two adjacent loci from its true value to evaluate these estimators. Since the ELS and BAT estimators work in three-point system, three-point recombination fractions were incorporated to two-point recombination fractions by using Tan and Fu [19] method. Simulation of codominant and dominant F_2_ populations and the ELS and BAT estimations of gamete frequencies in F_2_population were implemented by our R functions (Additional file 1, source code).

## Results

### Estimation of the frequencies of three-locus gametes in an F_2_ population

Since our ELS method for accurate estimation of the frequencies of three-locus gametes in a population with random union of gametes is based on dissection of phenotypes, for convenience, we start by presenting the BAT method of Tan and Fu [19].

### ELS estimation of frequencies of dominant marker gametes

Our study here is restricted to three biallelic dominant markers. We use *A* and *a*, *B* and *b*, *C* and *c* to represent two alleles at three loci where upper letters (A, B and C) stand for dominant alleles and lower letters (a, b and c) for recessive alleles. A triple-heterozygote individual via meiosis produces eight types of gametes at the three loci: *ABC, ABc, Abc, AbC, aBC, abC, aB*c and *abc*. Gametes *ABC* and *abc* are a pair of sister gametes on which two alleles at the all three loci are different and come from two different parents. Similarly, *Abc* and *aBC*, *abC* and *ABc*, *AbC* and *aBc* are also pairs of sister gametes. Two sister gametes theoretically have equal frequency in an F_2_population because no mutation, no migration, no gene conversion and no selection occur in such a random mating population. From the expectation that sister-gametes have equal frequencies, we have in an F_2_ population *f*(*ABC*) = *f*(*abc*) = *q*_1_, *f*(*ABC*) = *f*(*aBC*) = *q*_2_, *f*(*ABc*) = *f*(*aBC*) = *q*_3_, *f*(*AbC*) = *f*(*aBc*) = *q*_4_. These gamete frequencies are constrained by 2*q*_1_ + 2*q*_2_ + 2*q*_3_ + 2*q*_4_ = 1. The individuals in the population can be classified into four categories: category 0 in which all individuals possess 0 dominant locus, that is, all individuals have three recessive loci; categories 1, 2 and 3 in which all individuals have respectively only one, two and three homozygous or heterozygous dominant loci. To accurately estimate gamete frequencies, we dissect a phenotype into different zygote types (genotypes) in each category using sister gametes. In category 1, for example, *aabbC*_ has only locus c with one or two dominant alleles. Therefore it can be dissected into three zygote types:

1a $$ aabbC\_\to \Big\{{\displaystyle \begin{array}{l} aabbCC\to {(abC)}^2\kern1.00em :\kern1.40em {\left(f(abC)\right)}^2={q}_3^2\\ {} aabbCc\to (abC)(abc):f(abC)f(abc)={q}_3{q}_1\\ {} aabbcC\to (abc)(abC):f(abc)f(abC)={q}_1{q}_3\end{array}}\operatorname{}. $$

Phenotypes *aaB_cc* and *A_bbcc* are dissected in a similar fashion. Category 2 also has three phenotypes and each of them can be dissected into four zygote types that are comprised of five pairs of sister gametes. For instance, phenotype type *A*_*B*_*cc* can be dissected into

1b $$ A\_B\_ cc\to \Big\{{\displaystyle \begin{array}{l} AABBcc\to (ABc)(ABc):f(ABc)f(ABc)=\kern0.5em {q}_3^2\kern1.00em \\ {} AaBbcc\to (ABc)(abc):f(ABc)f(abc)=2{q}_3{q}_1\kern1.00em \\ {} AABbcc\to (ABc)(Abc):f(ABc)f(Abc)=2{q}_3{q}_2\kern1.00em \\ {} AaBBcc\to (ABc)(aBc):f(ABc)f(aBc)=2{q}_3{q}_4\\ {} AaBbcc\to (Abc)(aBc):f(Abc)f(aBc)=2{q}_2{q}_4\end{array}}\operatorname{}. $$

Category 3 has only one phenotype. The phenotype is comprised of 8 zygote types (genotypes) and therefore it is not useful for estimate of gamete frequencies. We use *Q*_1_, *Q*_2_, *Q*_3_, *Q*_4_, *Q*_5_, *Q*_6_, and *Q*_7_ to respectively represent the frequency expectations of phenotypes *aabbcc*, *aabbC*_, *aaB*_*cc*, *A*_*bbcc*, *A*_*B*_*cc*, *A*_*bbC*_, and *aaB*_*C*_ in a population. The frequency of phenotype *aabbcc* is

2 $$ f(aabbcc)={Q}_1={q}_1^2 $$

The other 6 phenotypes have their frequencies:

3 $$ \Big\{{\displaystyle \begin{array}{c}f\left( aabbC\_\right)={Q}_2={q}_3^2+2{q}_1{q}_3\\ {}f\left( aaB\_ cc\right)={Q}_3={q}_4^2+2{q}_1{q}_4\\ {}f\left(A\_ bbcc\right)={Q}_4={q}_2^2+2{q}_1{q}_2\end{array}}\operatorname{}. $$

4 $$ \Big\{{\displaystyle \begin{array}{c}f\left(A\_B\_ cc\right)={Q}_5={q}_3^2+2{q}_1{q}_3+2\left({q}_3{q}_2+{q}_3{q}_4+{q}_2{q}_4\right)\\ {}f\left(A\_ bbC\_\right)={Q}_6={q}_4^2+2{q}_1{q}_4+2\left({q}_3{q}_2+{q}_3{q}_4+{q}_2{q}_4\right)\\ {}f\left( aaB\_C\_\right)={Q}_7={q}_2^2+2{q}_1{q}_2+2\left({q}_3{q}_2+{q}_3{q}_4+{q}_2{q}_4\right)\end{array}}\operatorname{}. $$

Using *Q* = 2 (*q*_2_ *q*_3_ + *q*_2_ *q*_4_ + *q*_3_ *q*_4_), Eq. (4) is simplified as

5 $$ \Big\{{\displaystyle \begin{array}{c}{Q}_5={Q}_2+Q\\ {}{Q}_6={Q}_3+Q\\ {}{Q}_7={Q}_4+Q\end{array}}\operatorname{}. $$

Estimates of *q*_1_ ,  …  , *q*_4_ can be obtained from the above sets of equations by replacing *Q*_*k*_ with their observed frequencies where k = 1, 2,…,7 for 7 phenotypes. Theoretically, eqs. (1) and (3) are sufficient to make solutions for the frequencies of four types of gametes. However, Eq. (5) can be used to further minimize noise in the observed frequencies. That is, *Q*_2_, *Q*_3_,and *Q*_4_ can be alternatively estimated as

6 $$ \left\{\begin{array}{c}\hfill {\widehat{Q}}_2^{\#}={\widehat{Q}}_5-\widehat{Q}=0.25-\left({\widehat{Q}}_1+{\widehat{Q}}_6+{\widehat{Q}}_7\right)\hfill \\ {}\hfill {\widehat{Q}}_3^{\#}={\widehat{Q}}_6-\widehat{Q}=0.25-\left({\widehat{Q}}_1+{\widehat{Q}}_5+{\widehat{Q}}_7\right)\hfill \\ {}\hfill {\widehat{Q}}_4^{\#}={\widehat{Q}}_7-\widehat{Q}=0.25-\left({\widehat{Q}}_1+{\widehat{Q}}_5+{\widehat{Q}}_6\right)\hfill \end{array}\right. $$

where *Q* = *Q*_5_ + *Q*_6_ + *Q*_7_ + *Q*_1_ − 0.25 [19]. It implicates that *Q*_2_, *Q*_3_, and *Q*_4_ can also be estimated from the estimated frequencies of *Q*_1_, *Q*_5_, *Q*_6_, and *Q*_7_. Thus, we can combine the two sets of estimates of *Q*_2_, *Q*_3_, and *Q*_4_ into one set:

7 $$ \left\{\begin{array}{c}\hfill {\widehat{Q}}_2^{\ast }=\frac{1}{a_2+{b}_2}\left({a}_2{\widehat{Q}}_2+{b}_2{\widehat{Q}}_2^{\#}\right)\hfill \\ {}\hfill {\widehat{Q}}_3^{\ast }=\frac{1}{a_3+{b}_3}\left({a}_3{\widehat{Q}}_3+{b}_3{\widehat{Q}}_3^{\#}\right)\hfill \\ {}\hfill {\widehat{Q}}_4^{\ast }=\frac{1}{a_4+{b}_4}\left({a}_4{\widehat{Q}}_4+{b}_4{\widehat{Q}}_4^{\#}\right)\hfill \end{array}\right. $$

where *a*_*k*_ and *b*_*k*_ are weights of ${\widehat{Q}}_k$ and ${\widehat{Q}}_k^{\#}$, respectively, where *k* = 2, 3, and 4. ${\widehat{Q}}_k$ and ${\widehat{Q}}_k^{\#}$ are respectively estimates of *Q*_*k*_ and ${Q}_k^{\#}$. In general case, *a*_*k*_ = *b*_*k*_ (see Additional file 3: Appendix B). An alternative method for weighting is ${a}_k={\widehat{Q}}_k/\left({\widehat{Q}}_k+{\widehat{Q}}_k^{\#}\right)$ and *b*_*k*_ = 1 − *a*_*k*_. When the sample is small, it is likely that ${\widehat{Q}}_k^{\#}\le$ 0 or ${\widehat{Q}}_k=$ 0. In such a case, one can set *a*_*k*_ = 1 and *b*_*k*_ = 0 for ${\widehat{Q}}_k^{\#}\le$ 0, or *a*_*k*_ = 0 and *b*_*k*_ = 1 for ${\widehat{Q}}_k^{\#}>$ 0 and ${\widehat{Q}}_k$ = 0. Since ${Q}_2={q}_3^2+2{q}_1{q}_3+{q}_1^2-{q}_1^2={\left({q}_3+{q}_1\right)}^2-{q}_1^2$, *q*_3_ can be given by

8a $$ {q}_3=\sqrt{Q_2+{Q}_1}-\sqrt{Q_1}. $$

Similarly,

8b $$ {q}_2=\sqrt{Q_4+{Q}_1}-\sqrt{Q_1}, $$

8c $$ {q}_4=\sqrt{Q_3+{Q}_1}-\sqrt{Q_1}. $$

*Q*_1_, *Q*_2_, *Q*_3_, and *Q*_4_ are respectively estimated by ${\widehat{Q}}_1$, ${\widehat{Q}}_2^{\ast }$, ${\widehat{Q}}_3^{\ast }$, ${\widehat{Q}}_4^{\ast }$, therefore *q*_3_, *q*_2_, *q*_4_, and *q*_1_ are respectively estimated by

9a $$ {\hat{q}}_3=\sqrt{{\hat{Q}}_2+{\hat{Q}}_1}-\sqrt{{\hat{Q}}_1}, $$

9b $$ {\hat{q}}_2=\sqrt{{\hat{Q}}_4+{\hat{Q}}_1}-\sqrt{{\hat{Q}}_1}, $$

9c $$ {\hat{q}}_4=\sqrt{{\widehat{Q}}_3^{\ast }+{\hat{Q}}_1}-\sqrt{{\hat{Q}}_1}, $$

9d $$ {\hat{q}}_1=\sqrt{{\hat{Q}}_1}. $$

In Eq. (9), accurate estimation of *q*_1_ is a key contribution to accurate estimations of *q*_2_, *q*_3_, and *q*_4_. Equations (3) and (4) show that *Q*_2_ ~ *Q*_7_ can also provide information of solution to *q*_1_. But it is impossible to directly obtain a solution for *q*_1_ from *Q*_2_ ~ *Q*_7_. To estimate *q*_1_ from *Q*_1_ ~ *Q*_7_, we here proposed a seeking method, named “expectation least square” (ELS) method.

Similar to the EM method [11, 25, 28, 29], the ELS method also consists of two steps. The first step is the expectation step, denoted by E-step, and the second step is the least-square step, denoted by LS-step. *q*_1_ is initialized to be ${\widehat{q}}_1^0=\sqrt{{\widehat{Q}}_1}$. We use ${\widehat{q}}_1^0$ to estimate *q*_2_, *q*_3_, and *q*_4_ and get ${\widehat{q}}_2^0$, ${\widehat{q}}_3^0$, and ${\widehat{q}}_4^0$ from Eqs. (9). Then, we calculate the expected values of *Q*_2_ ~ *Q*_7_ from Eqs. (3) ~ (4) with ${\widehat{q}}_2^0$, ${\widehat{q}}_3^0$, and ${\widehat{q}}_4^0$ . At iteration j, we realize E-step and LS-step to get ${\widehat{q}}_2^j$, ${\widehat{q}}_3^j$, and ${\widehat{q}}_4^j$:

#### E-step:

Calculate the expected values $E\left({Q}_2^j\right)$ ~ $E\left({Q}_7^j\right)$ of *Q*_2_ ~ *Q*_7_ by replacing ${\widehat{q}}_1^j$, ${\widehat{q}}_2^j$, ${\widehat{q}}_3^j$, and ${\widehat{q}}_4^j$ into Eqs. (3) ~ (4) where ${\widehat{q}}_2^j$, ${\widehat{q}}_3^j$, and ${\widehat{q}}_4^j$ are obtained by

$$ {\hat{q}}_2^j=\sqrt{Q_4^{\ast j}+{\left({\hat{q}}_1^j\right)}^2}-{\hat{q}}_1^j, $$

$$ {\hat{q}}_3^j=\sqrt{Q_2^{\ast j}+{\left({\hat{q}}_1^j\right)}^2}-{\hat{q}}_1^j, $$

$$ {\widehat{q}}_4^j=\sqrt{Q_3^{\ast j}+{\left({\widehat{q}}_1^j\right)}^2}-{\widehat{q}}_1^j $$

where

$$ {Q}_i^{\ast j}=\frac{1}{a+b}\left(a{\widehat{Q}}_i+{bQ}_i^{\#j}\right) $$

where *i* = 2 , …, 4 and ${Q}_i^{\#j}={\widehat{Q}}_{i+3}-E\left({Q}^{j-1}\right)$ where $E\left({Q}^{j-1}\right)=2\left({\widehat{q}}_2^{j-1}{\widehat{q}}_3^{j-1}+{\widehat{q}}_2^{j-1}{\widehat{q}}_4^{j-1}+{\widehat{q}}_3^{j-1}{\widehat{q}}_4^{j-1}\right)$.

#### LS-step:

Calculate square value using

10 $$ {S}_j^2=\sum_{i=2}^7{\left({\widehat{Q}}_i-E\left({Q}_i^j\right)\right)}^2. $$

Note that ${\widehat{q}}_1^j$ is a value we want to seek for, therefore, Eq. (10) does not contain ${\left({\widehat{Q}}_1-{EQ}_1^j\right)}^2$. As it is very difficult to directly get solutions for these four q-values from the derivative approach, we use an iteration approach to minimize square value:

11 $$ {\hat{q}}_1^{j-1}=\arg \min \left({S}_{j-1}^2,{S}_j^2\right). $$

Use ${\widehat{q}}_1^j={\widehat{q}}_1^{j-1}\pm \varDelta$ to calculate ${\widehat{q}}_2^j$, ${\widehat{q}}_3^j$, and ${\widehat{q}}_4^j$ where j is the jth iteration, *j* = 1 , …, and *Δ* is specified with a very small value. Here our algorithm to realize LS-step is

If $$ {S}_j^2>{S}_{j-1}^2 $$, then

if $$ {\widehat{q}}_1^j>{\widehat{q}}_1^{j-1} $$, then $$ {\hat{q}}_1^j={\hat{q}}_1^{j-1}-\varDelta $$,

otherwise, $$ {\widehat{q}}_1^j={\widehat{q}}_1^{j-1}+\varDelta $$

else if ${S}_j^2<{S}_{j-1}^2$, then

if $$ {\widehat{q}}_1^j>{\widehat{q}}_1^{j-1} $$, then $$ {\widehat{q}}_1^j={\widehat{q}}_1^{j-1}+\varDelta $$,

otherwise, $$ {\widehat{q}}_1^j={\widehat{q}}_1^{j-1}-\varDelta $$.

Note that there are not ${S}_j^2={S}_{j-1}^2$ and ${\widehat{q}}_1^j={\widehat{q}}_1^{j-1}$ in this algorithm. The iteration will stop at ${S}_j^2\le t$ where *t* is a given tolerant value. Once the final estimate (${\widehat{q}}_1^f$) of *q*_1_ is found at a given tolerant value where *j* = *f*, the final estimates of *q*_2_, *q*_3_, and *q*_4_ are obtained. Then we let ${\widehat{q}}_1={\widehat{q}}_1^f$, ${\widehat{q}}_2={\widehat{q}}_2^f$, ${\widehat{q}}_3={\widehat{q}}_3^f$, and ${\widehat{q}}_4={\widehat{q}}_4^f$ .

### BAT for estimation of the frequencies of codominant marker gametes in F_2_ population

To avoid confusing notations in codominant loci with those in dominant loci, we let 0 and 1 code for homozygote from two parents, respectively, and 2 code for heterozygote at a locus. Since homozygote and heterozygote at three loci can be recognized, most of zygotes are informative for estimation of the frequencies of four pairs of sister gametes. We still assume that the sister-gametes have equal frequencies, that is, *q*_1_ = *f*(111) = *f*(000), *q*_2_ = *f*(100) = *f*(011), *q*_3_ = *f*(110) = *f*(001), *q*_4_ = *f*(101) =*f*(010) in F_2_ population. Here these complementary zygote type pairs are listed as follows:

$$ \mathrm{Zygote}\  \mathrm{gamete}\  \mathrm{frequency}\  \mathrm{expected}\  \mathrm{Zygote}\  \mathrm{gamete}\  \mathrm{frequency}\  \mathrm{expected} $$

$$ 111,\kern0.5em 000\to \left\{\begin{array}{c}\hfill (111)(111):{q}_1^2\hfill \\ {}\hfill (000)(000):{q}_1^2\hfill \end{array}\right.,{\displaystyle \begin{array}{cc}\hfill 100,\hfill & \hfill 011\hfill \end{array}}\to \left\{\begin{array}{c}\hfill (100)(100):{q}_2^2\hfill \\ {}\hfill (011)(011):{q}_2^2\hfill \end{array}\right., $$

$$ 110,\kern0.5em 001\to \left\{\begin{array}{c}\hfill (110)(110):{q}_3^2\hfill \\ {}\hfill (001)(001):{q}_3^2\hfill \end{array}\right.,{\displaystyle \begin{array}{cc}\hfill 101,\hfill & \hfill 010\hfill \end{array}}\to \left\{\begin{array}{c}\hfill (101)(101):{q}_4^2\hfill \\ {}\hfill (010)(010):{q}_4^2\hfill \end{array}\right., $$

$$ 200,\kern0.5em 211\to \left\{\begin{array}{c}\hfill (000)(100):2{q}_1{q}_2\hfill \\ {}\hfill (111)(011):2{q}_1{q}_2\hfill \end{array}\right.,{\displaystyle \begin{array}{cc}\hfill 112,\hfill & \hfill 002\hfill \end{array}}\to \left\{\begin{array}{c}\hfill (000)(001):2{q}_1{q}_3\hfill \\ {}\hfill (111)(110):2{q}_1{q}_3\hfill \end{array}\right., $$

$$ 121,\kern0.5em 020\to \left\{\begin{array}{c}\hfill (000)(010):2{q}_1{q}_4\hfill \\ {}\hfill (111)(101):2{q}_1{q}_4\hfill \end{array}\right.,{\displaystyle \begin{array}{cc}\hfill 021,\hfill & \hfill 120\hfill \end{array}}\to \left\{\begin{array}{c}\hfill (011)(001):2{q}_2{q}_3\hfill \\ {}\hfill (110)(100):2{q}_2{q}_3\hfill \end{array}\right., $$

$$ 102,\kern0.5em 012\to \left\{\begin{array}{c}\hfill (100)(101):2{q}_2{q}_4\hfill \\ {}\hfill (011)(010):2{q}_2{q}_4\hfill \end{array}\right.,{\displaystyle \begin{array}{cc}\hfill 201,\hfill & \hfill 210\hfill \end{array}}\to \left\{\begin{array}{c}\hfill (001)(101):2{q}_3{q}_4\hfill \\ {}\hfill (110)(010):2{q}_3{q}_4\hfill \end{array}\right., $$

$$ {\displaystyle \begin{array}{c}\hfill 122\kern0.36em \to \left\{\begin{array}{c}\hfill (111)(100):2{q}_1{q}_2\hfill \\ {}\hfill (110)(101):2{q}_3{q}_4\hfill \end{array}\right.,\hfill \\ {}\hfill 022\to \left\{\begin{array}{c}\hfill (000)(011):2{q}_1{q}_2\hfill \\ {}\hfill (001)(010):2{q}_3{q}_4\hfill \end{array}\right.,\hfill \end{array}}{\displaystyle \begin{array}{c}\hfill 221\to \left\{\begin{array}{c}\hfill (111)(001):2{q}_1{q}_3\hfill \\ {}\hfill (011)(101):2{q}_2{q}_4\hfill \end{array}\right.,\hfill \\ {}\hfill 220\to \left\{\begin{array}{c}\hfill (000)(110):2{q}_1{q}_3\hfill \\ {}\hfill (100)(010):2{q}_2{q}_4\hfill \end{array}\right.,\hfill \end{array}} $$

$$ 212\to \left\{\begin{array}{c}\hfill (111)(010):2{q}_1{q}_4\hfill \\ {}\hfill (110)(011):2{q}_2{q}_3\hfill \end{array}\right.,202\to \left\{\begin{array}{c}\hfill (000)(101):2{q}_1{q}_4\hfill \\ {}\hfill (100)(001):2{q}_2{q}_3\hfill \end{array}\right.. $$

Let *P*_1_, *P*_2_, *P*_3_ and *P*_4_ represent the frequencies of complementary homozygote types (111/000), (100/011), (110/001), and (101/010) in each of which all three loci are homozygous; let *P*_12_, *P*_13_, *P*_14_, *P*_23_, *P*_24_, and *P*_34_ be the frequencies of complementary two-locus homozygote types (200/211), (002/112), (121/020), (021/120), (102/012), and (201/210) in each of which only one locus are heterozygous and let *P*_1234_, *P*_1324_, *P*_1423_ be the frequencies of complementary one-locus homozygote types (122/022), (221/220) and (212/202) in each of which two loci are heterozygous. Then, ${P}_1=2{q}_1^2$, ${P}_2=2{q}_2^2$, ${P}_3=2{q}_3^2$, ${P}_4=2{q}_4^2$, *P*_12_ = 4*q*_1_ *q*_2_, *P*_13_ = 4*q*_1_ *q*_3_, *P*_14_ = 4*q*_1_ *q*_4_, *P*_23_ = 4*q*_2_ *q*_3_, *P*_24_ = 4*q*_2_ *q*_4_, *P*_34_ = 4*q*_3_ *q*_4_, *P*_1234_ = 4*q*_1_ *q*_2_ + 4*q*_3_ *q*_4_, *P*_1324_ = 4*q*_1_ *q*_3_ + 4*q*_2_ *q*_4_, *P*_1423_ = 4*q*_1_ *q*_4_ + 4*q*_2_ *q*_3_. From the zygote type pair list above, we find that the frequencies of these 12 pairs of zygote types can constitute two sets of 6 binomial equations:

12a $$ {Q}_{12}^1=\frac{1}{2}\left({P}_1+{P}_{12}+{P}_2\right)={q}_1^2+2{q}_1{q}_2+{q}_2^2={\left({q}_1+{q}_2\right)}^2, $$

12b $$ {Q}_{13}^1=\frac{1}{2}\left({P}_1+{P}_{13}+{P}_3\right)={q}_1^2+2{q}_1{q}_3+{q}_3^2={\left({q}_1+{q}_3\right)}^2, $$

12c $$ {Q}_{14}^1=\frac{1}{2}\left({P}_1+{P}_{14}+{P}_4\right)={q}_1^2+2{q}_1{q}_4+{q}_4^2={\left({q}_1+{q}_4\right)}^2, $$

12d $$ {Q}_{23}^1=\frac{1}{2}\left({P}_2+{P}_{23}+{P}_3\right)={q}_2^2+2{q}_2{q}_3+{q}_3^2={\left({q}_2+{q}_3\right)}^2, $$

12e $$ {Q}_{24}^1=\frac{1}{2}\left({P}_2+{P}_{24}+{P}_4\right)={q}_2^2+2{q}_2{q}_4+{q}_4^2={\left({q}_2+{q}_4\right)}^2, $$

12f $$ {Q}_{34}^1=\frac{1}{2}\left({P}_3+{P}_{34}+{P}_4\right)={q}_3^2+2{q}_3{q}_4+{q}_4^2={\left({q}_3+{q}_4\right)}^2 $$

13a $$ {Q}_{12}^2=\frac{1}{2}\left({P}_1+{P}_{1234}-{P}_{34}+{P}_2\right)={q}_1^2+2{q}_1{q}_2+{q}_2^2={\left({q}_1+{q}_2\right)}^2, $$

13b $$ {Q}_{13}^2=\frac{1}{2}\left({P}_1+{P}_{1324}-{P}_{24}+{P}_3\right)={q}_1^2+2{q}_1{q}_3+{q}_3^2={\left({q}_1+{q}_3\right)}^2, $$

13c $$ {Q}_{14}^2=\frac{1}{2}\left({P}_1+{P}_{1423}-{P}_{23}+{P}_4\right)={q}_1^2+2{q}_1{q}_4+{q}_4^2={\left({q}_1+{q}_4\right)}^2, $$

13d $$ {Q}_{23}^2=\frac{1}{2}\left({P}_2+{P}_{1423}-{P}_{14}+{P}_3\right)={q}_2^2+2{q}_2{q}_3+{q}_3^2={\left({q}_2+{q}_3\right)}^2, $$

13e $$ {Q}_{24}^2=\frac{1}{2}\left({P}_2+{P}_{1324}-{P}_{13}+{P}_4\right)={q}_2^2+2{q}_2{q}_4+{q}_4^2={\left({q}_2+{q}_4\right)}^2, $$

13f $$ {Q}_{34}^2=\frac{1}{2}\left({P}_3+{P}_{1234}-{P}_{12}+{P}_4\right)={q}_3^2+2{q}_3{q}_4+{q}_4^2={\left({q}_3+{q}_4\right)}^2. $$

We use arithmetic mean to get frequencies of these zygote types in F_2_ population:

14 $$ {Q}_{ij}=\left({a}_{ij}{Q}_{ij}^1+{b}_{ij}{Q}_{ij}^2\right)={\left({q}_i+{q}_j\right)}^2, $$

where ${a}_{ij}={\widehat{Q}}_{ij}^1/\left({\widehat{Q}}_{ij}^1+{\widehat{Q}}_{ij}^2\right)$ and *b*_*ij*_ = 1 − *a*_*ij*_. $\left({a}_{ij}{Q}_{ij}^1+{b}_{ij}{Q}_{ij}^2\right)={a}_{ij}{\left({q}_i+{q}_j\right)}^2$ +*b*_*ij*_(*q*_*i*_ + *q*_*j*_)^2^= (*a*_*ij*_ + *b*_*ij*_) (*q*_*i*_ + *q*_*j*_)^2^ = (*q*_*i*_ + *q*_*j*_)^2^ where *i* and *j* are gamete types *i* and *j* (*i* = 1, 2, 3 and *j* = 2, 3, 4 and *i* ≠ *j*). Thus, the frequencies of four types of non-sister gametes in a codominant three-locus system in an F_2_population are easily and fast estimated by

15a $$ {\widehat{q}}_1=\frac{1}{2}\left(\frac{\sqrt{{\widehat{Q}}_{12}}+\sqrt{{\widehat{Q}}_{13}}+\sqrt{{\widehat{Q}}_{14}}-\left(\sqrt{\frac{1}{2}{\widehat{P}}_2}+\sqrt{\frac{1}{2}{\widehat{P}}_3}+\sqrt{\frac{1}{2}{\widehat{P}}_4}\right)}{3}+\sqrt{\frac{{\widehat{P}}_1}{2}}\right), $$

15b $$ {\widehat{q}}_2=\frac{1}{2}\left(\frac{\sqrt{{\widehat{Q}}_{12}}+\sqrt{{\widehat{Q}}_{23}}+\sqrt{{\widehat{Q}}_{24}}-\left(\sqrt{\frac{1}{2}{\widehat{P}}_1}+\sqrt{\frac{1}{2}{\widehat{P}}_3}+\sqrt{\frac{1}{2}{\widehat{P}}_4}\right)}{3}+\sqrt{\frac{{\widehat{P}}_2}{2}}\right), $$

15c $$ {\widehat{q}}_3=\frac{1}{2}\left(\frac{\sqrt{{\widehat{Q}}_{13}}+\sqrt{{\widehat{Q}}_{23}}+\sqrt{{\widehat{Q}}_{34}}-\left(\sqrt{\frac{1}{2}{\widehat{P}}_1}+\sqrt{\frac{1}{2}{\widehat{P}}_2}+\sqrt{\frac{1}{2}{\widehat{P}}_4}\right)}{3}+\sqrt{\frac{{\widehat{P}}_3}{2}}\right), $$

15d $$ {\widehat{q}}_4=\frac{1}{2}\left(\frac{\sqrt{{\widehat{Q}}_{14}}+\sqrt{{\widehat{Q}}_{24}}+\sqrt{{\widehat{Q}}_{34}}-\left(\sqrt{\frac{1}{2}{\widehat{P}}_1}+\sqrt{\frac{1}{2}{\widehat{P}}_2}+\sqrt{\frac{1}{2}{\widehat{P}}_3}\right)}{3}+\sqrt{\frac{{\widehat{P}}_4}{2}}\right), $$

where ${\widehat{Q}}_{ij}$ and ${\widehat{P}}_k$ are respective estimates of *Q*_*ij*_ and *P*_*k*_ in F_2_ population where *k* = 1,…,4 denote gamete types 1, …, 4.

A modified BAT method (BAT II) for estimating the frequencies of eight gamete types without assumption that the sister gametes have equal frequencies in any generation population is given in Additional file 2, Appendix A.

### Estimation of recombination fractions

Since these four *q*s are estimated separately, sum of them does not always satisfy a constraint of ${\widehat{q}}_1+{\widehat{q}}_2+{\widehat{q}}_3+{\widehat{q}}_4=0.5$. For this reason, we normalize our estimates as

16 $$ \left\{\begin{array}{cc}\hfill {p}_1=\frac{{\widehat{q}}_1}{2\widehat{q}},\hfill & \hfill {p}_3=\frac{{\widehat{q}}_3}{2\widehat{q}}\hfill \\ {}\hfill {p}_2=\frac{{\widehat{q}}_2}{2\widehat{q}},\hfill & \hfill {p}_4=\frac{{\widehat{q}}_4}{2\widehat{q}}\hfill \end{array}\right.. $$

For three linked loci, the frequencies of the four gamete pairs can be used to find the double crossover types by distinguishing coupling phase from repulsion phase between loci. For example, for an order a-b-c of the three loci a, b and c, *p*_4_ is determined to be the frequency of double crossover types if its value is the smallest and/or *p*_1_ is the largest, which are produced at three coupling loci or *p*_1_ is found to be the frequency of double crossover types if its value is the smallest and/or *p*_4_ is the largest, which are formed at loci a and c in coupling phase and locus b in repulsion phase. In a similar way, we can also define *p*_3_ or *p*_2_ as the frequency of double crossover types.

If *p*_4_ is frequency of double crossover types, then the recombination fractions between loci *a* and *b*, between loci *b* and *c*, and between loci *a* and *c* can be estimated by

17 $$ \left\{\begin{array}{c}\hfill {r}_{ab}=2\left({p}_3+{p}_4\right)\hfill \\ {}\hfill {r}_{bc}=2\left({p}_2+{p}_4\right)\hfill \\ {}\hfill {r}_{ac}=2\left({p}_2+{p}_3\right)\hfill \end{array}\right.. $$

For the linkage orders a-c-b and b-a-c, the recombination fractions between loci are also estimated in a similar way.

In the repulsion phase, the linkage *a-b-c* order of three loci determines *p*_1_ to be the frequency of double crossover types, so estimates of recombination fractions between loci *a* and *b*, between loci *b* and *c*, and between loci *a* and *c* are

18 $$ \left\{\begin{array}{c}\hfill {r}_{ab}=2\left({p}_2+{p}_1\right)\hfill \\ {}\hfill {r}_{bc}=2\left({p}_3+{p}_1\right)\hfill \\ {}\hfill {r}_{ac}=2\left({p}_2+{p}_3\right)\hfill \end{array}\right.. $$

For the linkage orders b-a-c and a-c-b, the recombination fractions between three loci in the repulsion phase can be estimated in this way.

*r*_*ab*_ , *r*_*bc*_ , and *r*_*ac*_ are simple notations of three recombination fractions in a triple. However, when *n* markers on a chromosome or a fragment are genotyped, it is difficult to use these notations of three recombination frequencies to denote recombination fractions in *n*(*n* − 1)(*n* − 2)/6 triples. To notate recombination fractions in multiple triples, we let *r*_*ab*_ = *r*_*abc*_ where *c* is referred to as a reference marker for recombination fraction between markers *a* and *b*, *r*_*ac*_ = *r*_*acb*_ where *b* as reference marker for that between loci *a* and *c*, and *r*_*bc*_ = *r*_*bca*_ where *a* as reference locus for that between markers *b* and c, in a three-locus system consisting of markers *a*, *b*, and *c* [19]. In more general fashion, we denote *i* for the first marker, *j* for the second maker, and *k* for the last marker. Thus, the rest *n* − 2 markers are combined with loci *i* and *j* into *n* − 2 three-points, therefore, there are *n* − 2 estimates of the recombination fraction between markers *i* and *j*. Hence estimate of recombination fraction between loci *i* and *j* is given by Tan and Fu’s method [19]:

19 $$ {\theta}_{ij}=\frac{1}{n-2}\sum_{k=1}^{n-2}{r}_{ij k}. $$

### Practical examples

Here we used RFLP (restriction fragment length polymorphisms) data of 333 F_2_ mice from MAPMAKER/EXP (version 3.0b), LANDER et al. [13] to illustrate performances of our ELS and BAT methods to estimate recombination fractions between dominant and codominant loci. RFLP markers are codominant markers. In genotype data of 333 F2 mice, “A” stands for homozygote A (two alleles from parent A), “H” for heterozygote H (an allele from parent A and the other from parent B), and “B” for homozygote B (two alleles from parent B). We arbitrarily selected 6 codominant markers from the original genotype data. To evaluate our ELS algorithm, we converted the codominant genotype data into dominant genotype data by changing B to H. For convenience, we used arabic digits (1, 2,…,6) to label these six markers: marker 1, marker 2, …, marker 6. Sometime we also used locus 1, locus 2, …, locus 6 to mark these six marker loci. The frequencies of 20 non-sister gametes were estimated by respectively performing ELS on the dominant data and BAT on the codominant genotype data, normalized by using Eq. (16) and the results are summarized in Tables 1 and 2. For the ELS estimation, three non-sister gametes containing loci 4 and 6 (146, 246 and 346) fitted well the ratio of 1:1:1:1 (Chi-square test *p*-value >0.084, Table 1), indicating that loci 4 and 6 are unlinked to loci 1, 2 and 3. In addition, the frequencies of gametes 256, 356, and 345 also fitted the ratio of 1:1:1:1 with *p*-value ≥ 0.063 (Chi-square test, Table 1), but gametes 156, 245 and 145 had the ratios significantly deviating against 1:1:1:1 (Chi-square test *p*-value <0.0212, Table 1), we could infer that locus 5 was linked to loci 1 but independent of locus 3 and unascertained at locus 2. Thus, we definitely excluded loci 4 and 6 in the linkage. By using eqs. (17) – (19), the recombination fractions in four triples (123), (125), (135), and (235) were calculated by following the five given steps: the first step is to determine the linkage order of three loci in triple. For example, in triple (123), *p*_1_ = *f(abc) =* 0.208668 is the largest value while *p*_2_ = *f(Abc)* = 0.086162 is the smallest one, that is to say, gamete *Abc* is double crossover type and *abc* is parental type, so their order is 2(b)-1(a)-3(c). Step2 is to determine linkage phase: since gamete *bac* is parental type and *bAc* is double crossover type, gamete *BAC or bac* is couple phase. At step 3, we abstracted frequencies of gametes 123, 125, 135, 235 (Table 3) from Table 1. At step 4, recombination fractions between loci in a triple were estimated as

$$ {r}_{bac(213)}=2\left[f(Abc)+f(aBc)\right]=2\left(0.086162+0.11047\right)=0.39327 $$

$$ {r}_{acb(132)}=2\left[f(Abc)+f(abC)\right]=2\left(0.086162+0.09469\right)=0.36172 $$

$$ {r}_{bca(231)}=2\left[f(aBc)+f(abC)\right]=2\left(0.086162+0.09469\right)=0.41034 $$

**Table 1:** The ELS estimated frequencies of four nonsister gametes in 20 triplets of 6 dominant loci in 333 F2 mice^a^

locus			frequency of non-sister gamete				Chi-square test	
a	b	c	*p1 = f(abc)*	*p2 = f(Abc)*	*p4 = f(aBc)*	*p3 = f(abC)*	*p*-value	ratio
1	2	3	0.208668	0.086162	0.094698	0.110472	0.000339	
1	2	4	0.14751	0.087958	0.160866	0.103665	0.028	
1	2	5	0.200976	0.080676	0.108494	0.109854	0.00092	
1	2	6	0.192229	0.084408	0.126033	0.09733	0.0023	
1	3	4	0.140566	0.079252	0.181795	0.098387	0.0038	
1	3	5	0.209093	0.065783	0.12237	0.102753	0.00012	
1	3	6	0.16895	0.063323	0.165648	0.102079	0.0011	
1	4	5	0.173539	0.100396	0.079665	0.1464	0.0069	
1	4	6	0.16482	0.102771	0.113819	0.118591	0.0837	1:1:1:1
1	5	6	0.173447	0.07932	0.148645	0.098588	0.0059	
2	3	4	0.141958	0.088919	0.172784	0.096339	0.012	
2	3	5	0.202566	0.085775	0.112098	0.099561	0.00084	
2	3	6	0.139672	0.086032	0.16843	0.105866	0.0212	
2	4	5	0.173634	0.117152	0.085607	0.123607	0.0212	
2	4	6	0.10113	0.133668	0.133668	0.131535	0.3134	1:1:1:1
2	5	6	0.156859	0.096648	0.142758	0.103735	0.0634	1:1:1:1
3	4	5	0.143582	0.120278	0.098137	0.138002	0.1954	1:1:1:1
3	4	6	0.105025	0.139707	0.129181	0.126086	0.3581	1:1:1:1
3	5	6	0.140841	0.109125	0.152351	0.097682	0.1028	1:1:1:1
4	5	6	0.146847	0.096392	0.156905	0.099856	0.0476	

a: The data came from MAPMAKER/EXP(3.0b) [27]

**Table 2:** The BAT estimated frequencies of nonsister gametes in 20 triplets of 6 codominant loci in 333 F2 mice^a^

*locus*			*frequency of non-sister gamete*				*Chi-square test*	
*a*	*b*	*c*	*p1 = f*(000)	*p2 = f*(100)	*p3 = f*(001)	*p4 = f*(010)	*p-value*	*ratio*
1	2	3	0.242929	0.066568	0.080146	0.110358	3.348e-07	
1	2	4	0.145838	0.08977	0.162134	0.102258	0.0291	
1	2	5	0.196094	0.091387	0.121051	0.091467	0.0017	
1	2	6	0.17224	0.104184	0.143308	0.080268	0.0098	
1	3	4	0.165099	0.068697	0.191983	0.074221	7.1334e-05	
1	3	5	0.222931	0.079297	0.147828	0.049944	1.0943e-06	
1	3	6	0.177615	0.065713	0.187929	0.068743	2.3780e-05	
1	4	5	0.158699	0.103969	0.114759	0.122573	0.1336	1:1:1:1
1	4	6	0.165089	0.113628	0.139128	0.082155	0.0249	
1	5	6	0.155874	0.091722	0.16263	0.089774	0.0121	
2	3	4	0.142565	0.093943	0.179432	0.08406	0.0050	
2	3	5	0.216411	0.069533	0.134853	0.079203	2.0404e-05	
2	3	6	0.160337	0.092614	0.172787	0.074262	0.0020	
2	4	5	0.172459	0.100044	0.105018	0.12248	0.0365	
2	4	6	0.154284	0.140079	0.121173	0.084464	0.0559	1:1:1:1
2	5	6	0.167782	0.072156	0.153072	0.10699	0.0057	
3	4	5	0.154314	0.118649	0.10895	0.118086	0.2051	1:1:1:1
3	4	6	0.144358	0.108635	0.131647	0.115359	0.3080	1:1:1:1
3	5	6	0.16738	0.053399	0.176627	0.102594	0.0002	
4	5	6	0.153613	0.092081	0.124467	0.129838	0.1092	1:1:1:1

a: The data came from MAPMAKER/EXP(3.0b) [27]

**Table 3:** The ELS estimated frequencies of nonsister gametes in triplets of dominant loci 1, 2, 3 and 5 in 333 F2 mice

locus			frequency of gamete			
*a*	*b*	*c*	*p1 = f(abc)*	*p2 = f(Abc)*	*p3 = f(abC)*	*p4 = f(aBc)*
1	2	3	0.208668	0.086162	0.094698	0.110472
1	2	5	0.200976	0.080676	0.108494	0.109854
1	3	5	0.209093	0.065783	0.12237	0.102753
2	3	5	0.202566	0.085775	0.112098	0.099561

Similarly, we also estimated the recombination fractions in triples (125), (135), and (235) (Table 3). Finally, the three-point estimates of the recombination fractions were incorporated into two-point estimates by applying Eq. (19) to the data in Table 4:

$$ {\theta}_{12}=\frac{r_{213}+{r}_{215}}{2}=\frac{0.393268+0.38106}{2}=0.387164, $$

$$ {\theta}_{13}=\frac{r_{135}+{r}_{132}}{2}=\frac{0.337072+0.36172}{2}=0.349396, $$

$$ {\theta}_{15}=\frac{r_{152}+{r}_{153}}{2}=\frac{0.37834+0.376306}{2}=0.377323, $$

$$ {\theta}_{23}=\frac{r_{231}+{r}_{235}}{2}=\frac{0.41034+0.370672}{2}=0.390506, $$

$$ {\theta}_{25}=\frac{r_{251}+{r}_{253}}{2}=\frac{0.436696+0.395746}{2}=0.416221, $$

$$ {\theta}_{35}=\frac{r_{351}+{r}_{352}}{2}=\frac{0.450246+0.423318}{2}=0.436782, $$

**Table 4:** The estimated recombination fractions between dominant loci in four triples

triple			Recombination fraction between loci		
*a*	*b*	*c*	*b-a*	*a-c*	*b-c*
1	2	3	0.39326	0.36172	0.41034
1	2	5	0.38106	0.37834	0.43669
1	3	5	0.33707	0.37631	0.45024
2	3	5	0.37067	0.39575	0.42332

Table 2 displays frequencies of codominant gametes estimated by our BAT method. It is clear to see that frequencies of gametes 145, 246, 345, 346, and 456 fitted well ratio of 1:1:1:1 with *p*-value ≥ 0.0559 (Chi-square test), however, the frequencies of gametes 156, 256 and 356 did not fit the ratio of 1:1:1:1 with *p*-value < 0.0121 (Chi-square test, Table 2), inferring that loci 4 and 6 are unlinked to loci 1, 2 and 3 but locus 5 could not be inferred to linked to them. Again, in codominant genotype data, locus 5 was still unascertained. Following the steps above, we obtained estimates of recombination fractions between these four loci (Table 5). Both ELS estimates of recombination fractions between dominant loci and BAT estimates between codominant loci show that locus 5 could not be tightly linked to any one of loci 1, 2 and 3. Loci 1, 2 and 3 could be determined to have linkage order of 2-1-3. Simulation data also showed that the codominant estimator had higher precision than the dominant estimator (see Simulation data section), suggesting that codominant markers indeed contain higher linkage information than dominant ones.

**Table 5:** Comparison between two estimators of recombination fractions between markers

two loci		the ELS estimate in dominant genotype data	the BAT estimate in codominant genotype data
1	2	0.387164	0.271317
1	3	0.349396	0.275955
1	5	0.377323	0.439563
2	3	0.390506	0.339240
2	5	0.416221	0.426574
3	5	0.436782	0.402158

### Simulation data

**Fig. 1:**
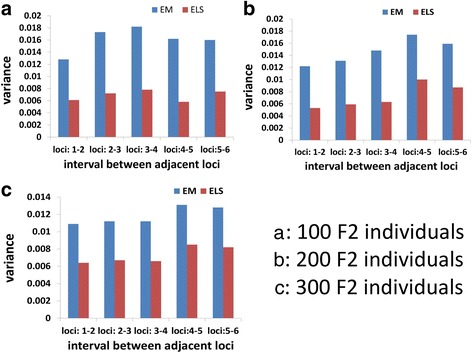
Variances of estimated recombination fractions between adjacent dominant loci in unknown linkage phase deviated from their respective true values Variance of estimated recombination fraction between adjacent dominant loci is given by simulating 100 estimates around true recombination fraction between adjacent loci. The variance here is equivalent to mean square error (MSE)

We performed simulation study to compare the two estimators of recombination fractions. We followed the simulation scheme of Tan and Fu [19]. Briefly, we set two linkage maps comprised of 6 dominant loci and 6 codominant loci, respectively. Five possible map distances 10, 15, 20, 25, and 30 cM (1 cM = 1%) were randomly assigned to the five adjacent intervals on these two linkage models with equal probability (see Methods for detail). The point process model [27] was used to generate F_2_ population. We did not consider recombination interference and linkage disequilibrium. Recombination fractions between adjacent loci in an unknown linkage phase (or say random phase) were estimated by the two-point EM [14, 23] and ELS estimators in 100 repeated samples of 100, 200, and 300 individuals drawn from the simulated F_2_ population. These two estimators were rated by the variance that quantifies deviation of estimated recombination fraction between two adjacent loci from its true value and is equivalent to mean squared error (MSE). For dominant markers, simulation shows that the ELS algorithm had much smaller variances in estimation of true recombination fractions between adjacent loci in samples of 100, 200 and 300 F_2_ individuals than two-point EM algorithm (Fig. 1). In Table 6, one can find that ELS had slightly higher probability of recovering true linkage maps of 6 loci than EM [14, 23] and BAT in the case of coupling phase and samples of 100 and 200 F_2_ individuals. When sample size reached 300 individuals, both ELS and EM recovered true coupling linkage maps with 100% probability and BAT also had 97.9% recovery rate. However, in unknown phase, ELS recovered true linkage maps of 6 loci with 23.4% probability in sample of 100 F_2_ individuals and reached 85% recovery rate in sample of 300 F_2_ individuals. By contrast, EM had very low recovery rate (23.4%) even when sample size was 300. Therefore, ELS performed much better than two-point EM algorithm in all given scenarios. An inexact comparison can be done between ELS and three-point EM algorithm of Lu et al. [30], Table 4 in Lu et al. showed that their three-point EM algorithms had 98.5% probability of finding the correct linkage map of three dominant markers in coupling phase from a sample of full-sib 100 individuals (corresponding to 100 F_2_ individuals), our ELS had 96.7% probability of recovering true linkage map of 6 dominant markers in coupling phase in 100 F_2_ individuals (Table 6). The probability to find a given linkage map will remarkably decrease as number of markers increases. So we can predict that the three-point EM algorithm would not have over 96.7% of the probability to find a given linkage map of 6 dominant markers. For the repulsion phase (or *trans* × *trans*), Lu et al.’s three-point EM algorithm had 99.5% probability of finding a correct linkage map of three markers in 100 full-sib individuals, which is higher than 98.6% in coupling phase. In theory, any EM algorithm should have much lower probability to find a given linkage order in repulsion phase than in coupling phase because the repulsion phase has much less linkage information content than the coupling phase [14, 26]. So, this result may be required to be confirmed in more simulations. Since Lu et al. did not implement simulation of random phase case and the repulsion phase is not random phase, the comparison cannot be made between the three-point EM and ELS algorithms in the random phase. For codominant markers, the BAT method performed with smaller variances than the two-point EM algorithm in the most cases. The results provided strong evidence for the conclusion that a method or algorithm based on three-point gametes can mitigate effect of low linkage information of repulsion phase on estimation of recombination fractions. Compared to the simulated results in Table 3 in [19], one can find that the ELS algorithm is better than the Tan and Fu’s BAT method. Table 3 in [19] showed that in case of unknown phase, the BAT method outperformed two-point EM.

**Table 6:** Efficiencies of estimators of recombination fractions in recovering the true linkage maps of 6 dominant loci in the case of random distance

Estimator		Linkage phase	Sample size		
			100	200	300
Two-point EM		CP	92.3	97.8	100.0
		UP	15.7	22.9	23.4
	ELS	CP	96.7	100.0	100.0
		UP	50.5	77.0	85.1
	BAT	CP	82.1	95.9	97.9
		UP	26.0	40.9	42.3

CP: Coupling phase and UP: unknown phase

## Discussion

Accurate estimation of recombination fractions is a key for mapping multiple markers. Therefore, powerful method for estimating recombination fractions is required. For dominant loci, the EM and ML methods have been verified to have low power to estimate frequencies of recombination between loci in repulsion phase [14, 19]. This is because the EM method cannot distinguish dominant homozygous genotypes from dominant heterozygous genotypes.

Compared to the EM algorithm, the ELS algorithm based on Tan and Fu’s method [19] has small bias for estimating recombination fractions between dominant loci on a chromosome in a larger F_2_ population due to the following reasons: (a) gamete analysis can effectively distinguish marker linkage phases; (b) accurately estimate *q*_1_, and (c) average of estimates of recombination fraction between two loci over all reference loci [Eq. (19)] effectively balances sampling error. Estimation of *q*_1_ is restriction of the Tan and Fu’s method. We here proposed iteration expectation-least square algorithm (ELS) to seek for accurate *q*_1_ estimation. This new algorithm is similar to expectation maximum algorithm and its statistical properties will be given by more simulation comparisons in elsewhere. In addition, importance for high efficiency of recombination fraction estimation is ${\widehat{Q}}_k^{\ast }$. ELS had much higher recovery rate by using ${\widehat{Q}}_k^{\ast }$ than by using ${\widehat{Q}}_k$ in both coupling and unknown phase (Table 6). Correlation analysis also indicated that ${\widehat{Q}}_k^{\ast }$ indeed has the linkage behavior similar to ${\widehat{Q}}_k$ (Additional file 3, Appendix B). Furthermore, we found that ${\widehat{Q}}_k^{\ast }$ obtained from a data set of 100 simulated samples of 100 F_2_ individuals has remarkably smaller variance than ${\widehat{Q}}_k$ (data not shown). To fully confirm that ${\widehat{Q}}_k^{\ast }$ is the optimal choice in our ELS method, ${\widehat{Q}}_k^{\ast }$ was taken into account where ${\widehat{Q}}_k^{\ast }$ = $\left({\widehat{Q}}_k+{\widehat{Q}}_k^o\right)/2$ if ${\widehat{Q}}_k^o$ >0, otherwise, ${\widehat{Q}}_k^{\ast }$ = ${\widehat{Q}}_k$. The simulated result showed that ~31% of linkage maps recovered true order of 6 dominant loci in samples of 100 F_2_ individuals, which is apparently lower than that by using ${\widehat{Q}}_k^{\ast }=\frac{1}{2}\left({\widehat{Q}}_k+{\widehat{Q}}_k^{\mathrm{o}}\right)$. For this reason, we chose ${\widehat{Q}}_k^{\ast }=\frac{1}{2}\left({\widehat{Q}}_k+{\widehat{Q}}_k^{\mathrm{o}}\right)$ in our ELS algorithm. Besides the ELS algorithm, average of recombination fraction between two loci over all reference loci greatly reduces noise of recombination fractions.

BATII given in Additional file 2, Appendix A, can be used to estimate frequencies of 8 codominant gamete types in any nature population because it does not require the assumption that the sister gametes have equal frequencies in a population. However, its estimation accuracy is not higher than the first BAT method in F_2_ population because sister-gametes really have equal frequencies and two-locus heterozygote types are not useful in the BATII. In a natural population, for example, human population, the frequencies of these gametes are not purely derived from recombination events but may be due to selection, genetic drift, migration and mutation. If, however, sister gametes are found to be equal in statistics, then these frequencies can still be used to inference recombination fractions between loci and recombination inference.

## Conclusions

Accurate estimation of recombination fractions between loci is given by methodologies developed for accurate estimation of gamete frequencies in a population. Analyses of simulated and real dominant and codominant data show that the ELS method proposed here is a powerful algorithm for accurate estimation of frequencies of gametes with unknown phase in dominant three-locus system in F_2_ population and BAT is a computationally efficient and powerful method for estimating frequencies of non-sister three-point codominant gametes.

## Additional files

Additional file 1: Source code: three R functions: BAT.R, ELS.R, simulatF2.R. (ZIP 4 kb)

Additional file 2: Appendix A. Binomial analysis of three-point method (BATII) is described in detail. BATII is used to estimate frequencies of sister gametes at codominant loci in natural populations. (DOCX 184 kb)

Additional file 3: Appendix B. A proof of a proposition that equal weights of two datasets combined into a dataset have maximum linkage information and minimum error for linkage analysis is given. (DOCX 41 kb)

## Abbreviations

BAT: Binomial analysis of three-point gametes
ELS: Expectation least square
EM: Expectation maximization
RFLP: Restriction fragment length polymorphism
