# Supplementary material for: New statistical methods for estimation of recombination fractions in F2 population
Source: BMC Bioinformatics. 2017 Oct 3;18(Suppl 11):404. doi: 10.1186/s12859-017-1804-8 (PMC5629630; doi:10.1186/s12859-017-1804-8)
Supplement: Supplementary file 2 — Appendix A. Binomial analysis of three-point method (BATII) is described in detail. BATII is used to estimate frequencies of sister gametes at codominant loci in natural populations. (DOCX 184 kb) [file 12859_2017_1804_MOESM2_ESM.docx]

**Appendix A: BAT II**

We still use 0 and 1 to code for homozygotes from one of two common ancestors, respectively, and 2 to code for heterozygotes at one locus. We define frequencies of gametes as$f(111)=p_{1},f(000)=q_{1}$,$f(011)=p_{2},f(100)=q_{2}$,$f(110)=p_{3},f(001)=q_{3}$,$f(101)=p_{4}$, and$f(010)=q_{4}$. Here these zygote types, gamete types and frequencies expected are listed as follows:

zygote gamete frequency expected zygote gamete frequency expected

$\begin{matrix} 111 & (111)(111) \end{matrix}\begin{matrix} & f(111)f(111)=p_{1}^{2} \end{matrix}$, $\begin{matrix} 000 & (000)(000) \end{matrix}\begin{matrix} & f(000)f(000)=q_{1}^{2} \end{matrix}$

$\begin{matrix} 011 & (011)(011) \end{matrix}\begin{matrix} & f(011)f(011) \end{matrix}=p_{2}^{2}$, $\begin{matrix} 100 & (100)(100) \end{matrix}\begin{matrix} & f(100)f(100)=q_{2}^{2} \end{matrix}$

$\begin{matrix} 110 & (110)(110)\begin{matrix} & f(110)f(110)=p_{3}^{2} \end{matrix} \end{matrix}$, $\begin{matrix} 001 & (001)(001)\begin{matrix} & f(001)f(001)=q_{3}^{2} \end{matrix} \end{matrix}$

$\begin{matrix} 101 & (101)(101) \end{matrix}\begin{matrix} & f(101)f(101)=p_{4}^{2} \end{matrix}$, $\begin{matrix} 101 & (010)(010)\begin{matrix} & f(010)f(010)=q_{4}^{2} \end{matrix} \end{matrix}$

$\begin{matrix} 211 & \left\{\begin{matrix} (111)(011) \\ (011)(111) \end{matrix}\right. \end{matrix}\begin{matrix} \\ \\ \end{matrix}\begin{matrix} f(111)f(011) \\ + \\ f(011)f(111) \end{matrix}=2p_{1}p_{2}$, $\begin{matrix} 200 & \left\{\begin{matrix} (000)(100) \\ (100)(000) \end{matrix}\right. \end{matrix}\begin{matrix} \\ \\ \end{matrix}\begin{matrix} f(000)f(100) \\ + \\ f(100)f(000) \end{matrix}=2q_{1}q_{2}$

$\begin{matrix} 112 & \left\{\begin{matrix} (110)(111) \\ (111)(110) \end{matrix}\right. \end{matrix}\begin{matrix} \\ \\ \end{matrix}\begin{matrix} f(110)f(111) \\ + \\ f(111)f(110) \end{matrix}=2p_{1}p_{3}$, $\begin{matrix} 002 & \left\{\begin{matrix} (000)(001) \\ (001)(000) \end{matrix}\right. \end{matrix}\begin{matrix} \\ \\ \end{matrix}\begin{matrix} f(000)f(001) \\ + \\ f(001)f(000) \end{matrix}=2q_{1}q_{3}$

$\begin{matrix} 121 & \left\{\begin{matrix} (111)(101) \\ (101)(111) \end{matrix}\right. \end{matrix}\begin{matrix} \\ \\ \end{matrix}\begin{matrix} f(111)f(101) \\ + \\ f(101)f(111) \end{matrix}=2p_{1}p_{4}$, $\begin{matrix} 020 & \left\{\begin{matrix} (000)(010) \\ (010)(000) \end{matrix}\right. \end{matrix}\begin{matrix} \\ \\ \end{matrix}\begin{matrix} f(000)f(010) \\ + \\ f(010)f(000) \end{matrix}=2q_{1}q_{4}$

$\begin{matrix} 021 & \left\{\begin{matrix} (011)(001) \\ (001)(011) \end{matrix}\right. \end{matrix}\begin{matrix} \\ \\ \end{matrix}\begin{matrix} f(011)f(001) \\ + \\ f(001)f(011) \end{matrix}=2p_{2}q_{3}$ $\begin{matrix} 120 & \left\{\begin{matrix} (100)(110) \\ (110)(100) \end{matrix}\right. \end{matrix}\begin{matrix} \\ \\ \end{matrix}\begin{matrix} f(100)f(110) \\ + \\ f(110)f(100) \end{matrix}=2q_{2}p_{3}$,

$\begin{matrix} 012 & \left\{\begin{matrix} (010)(011) \\ (011)(010) \end{matrix}\right. \end{matrix}\begin{matrix} \\ \\ \end{matrix}\begin{matrix} f(010)f(011) \\ + \\ f(011)f(010) \end{matrix}=2p_{2}q_{4}$,$\begin{matrix} 102 & \left\{\begin{matrix} (100)(101) \\ (101)(100) \end{matrix}\right. \end{matrix}\begin{matrix} \\ \\ \end{matrix}\begin{matrix} f(100)f(101) \\ + \\ f(101)f(100) \end{matrix}=2q_{2}p_{4}$

$\begin{matrix} 210 & \left\{\begin{matrix} (010)(110) \\ (110)(010) \end{matrix}\right. \end{matrix}\begin{matrix} \\ \\ \end{matrix}\begin{matrix} f(010)f(110) \\ + \\ f(110)f(010) \end{matrix}=2p_{3}q_{4}$ , $\begin{matrix} 201 & \left\{\begin{matrix} (001)(101) \\ (101)(001) \end{matrix}\right. \end{matrix}\begin{matrix} \\ \\ \end{matrix}\begin{matrix} f(001)f(101) \\ + \\ f(101)f(001) \end{matrix}=2q_{3}p_{4}$.

Let us denote respectively$P_{1}$,$P_{2}$, $P_{3}$, $P_{4}$as the frequencies of homozygote types (111), (011), (110), and (101) whose two gametes are the same and come from a common ancestor, $P_{1}=f(111)=p_{1}^{2}$,$P_{2}=f(011)=p_{2}^{2}$, $P_{3}=f(110)=p_{3}^{2}$, $P_{4}=f(101)=p_{4}^{2}$ and $Q_{1}$, $Q_{2}$, $Q_{3}$, and$Q_{4}$ as the frequencies of (000), (100), (001), and (010) whose two gametes are also the same but come from another common ancestor: $Q_{1}=f(00)=q_{1}^{2}$, $Q_{2}=f(100)=q_{2}^{2}$, $Q_{3}=f(001)=q_{3}^{2}$, and$Q_{4}=f(010)=q_{4}^{2}$ . Let$P_{12}$, $P_{13}$, $P_{14}$ be the frequencies of one-locus heterozygote types (211), (112), (121): $P_{12}=f(211)=2p_{1}p_{2}$, $P_{13}=f(112)=2p_{1}p_{3}$, $P_{14}=f(121)=2p_{1}p_{4}$ and $Q_{12}$, $Q_{13}$, and$Q_{14}$, the frequencies of one-heterozygote types (200), (002), (020) whose two gametes are different but derived from a common ancestor: $Q_{12}=f(200)=2q_{1}q_{2}$, $Q_{13}=f(002)=2q_{1}q_{3}$, and$Q_{14}=2q_{1}q_{4}$ and $P_{2}Q_{3}$, $P_{2}Q_{4}$, $P_{3}Q_{4}$, $Q_{2}P_{3}$, $Q_{2}P_{4}$, and$Q_{3}P_{4}$, frequencies of one-heterozygote types (021), (012), (210), (120), (102), and (201) whose two gametes are different and separately derived from two different ancestors: $P_{2}Q_{3}=f(021)=2p_{2}q_{3}$, $P_{2}Q_{4}=f(012)=2p_{2}q_{4}$, $P_{3}Q_{4}=f(210)=2p_{3}q_{4}$, $Q_{2}P_{3}=f(120)=2q_{2}p_{3}$, $Q_{2}P_{4}=f(102)=2q_{2}p_{4}$, and$Q_{3}P_{4}=f(201)=2q_{3}p_{4}$. From the zygote type list above, we find that the frequencies of these12 pairs of zygote types can constitute two sets of 6 binomial equations:

$S_{12}=(P_{1}+P_{12}+P_{2})=q_{1}^{2}+2q_{1}q_{2}+q_{2}^{2}=\left(q_{1}+q_{2}\right)^{2}$, (A1)

$S_{13}=(P_{1}+P_{13}+P_{3})=q_{1}^{2}+2q_{1}q_{3}+q_{3}^{2}=\left(q_{1}+q_{3}\right)^{2}$, (A2)

$S_{14}=(P_{1}+P_{14}+P_{4})=q_{1}^{2}+2q_{1}q_{4}+q_{4}^{2}=\left(q_{1}+q_{4}\right)^{2}$, (A3)

$S_{23}=(P_{2}+P_{2}Q_{3}+Q_{3})=p_{2}^{2}+2p_{2}q_{3}+q_{3}^{2}=\left(p_{2}+q_{3}\right)^{2}$, (A4)

, (A5)

(A6)

$T_{12}=(Q_{1}+Q_{12}+Q_{2})=q_{1}^{2}+2q_{1}q_{2}+q_{2}^{2}=\left(q_{1}+q_{2}\right)^{2}$, (A7)

$T_{13}=(Q_{1}+Q_{13}+Q_{3})=q_{1}^{2}+2q_{1}q_{3}+q_{3}^{2}=\left(q_{1}+q_{3}\right)^{2}$, (A8)

$T_{14}=(Q_{1}+Q_{14}+Q_{4})=q_{1}^{2}+2q_{1}q_{4}+q_{4}^{2}=\left(q_{1}+q_{4}\right)^{2}$, (A9)

, (A10)

, (A11)

. (A12)

Thus, the frequencies of 8 codominant gamete types in a population in any generation are easily and fast estimated by

, (A13)

, (A14)

, (A15)

(A16)

, (A17)

, (A18)

, (A19)

(A20)

where$\hat{P}_{1}$,$\hat{P}_{2}$, $\hat{P}_{3}$, $\hat{P}_{4}$, $\hat{Q}_{1}$, $\hat{Q}_{2}$, $\hat{Q}_{3}$, and$\hat{Q}_{4}$, $\hat{P}_{12}$, $\hat{P}_{13}$, $\hat{P}_{14}$, $\hat{Q}_{12}$, $\hat{Q}_{13}$, and$\hat{Q}_{14}$, $\hat{S}_{12}$, $\hat{S}_{13}$, $\hat{S}_{14}$, $\hat{S}_{23}$, $\hat{S}_{24}$, $\hat{S}_{34}$, $\hat{T}_{12}$, $\hat{T}_{13}$, $\hat{T}_{14}$, $\hat{T}_{23}$, $\hat{T}_{24}$, $\hat{T}_{34}$are respectively estimates of $P_{1}$,$P_{2}$, $P_{3}$, $P_{4}$, $Q_{1}$, $Q_{2}$, $Q_{3}$, and$Q_{4}$, $P_{12}$, $P_{13}$, $P_{14}$, $Q_{12}$, $Q_{13}$, and$Q_{14}$, S_12_, S_13_, S_14_, S_23_, S_24_, S_34_, T_12_,T_13_,T_14_, T_23_, T_24_ and T_34_.
