# Supplementary material for: New statistical methods for estimation of recombination fractions in F2 population
Source: BMC Bioinformatics. 2017 Oct 3;18(Suppl 11):404. doi: 10.1186/s12859-017-1804-8 (PMC5629630; doi:10.1186/s12859-017-1804-8)
Supplement: Supplementary file 3 — Appendix B. A proof of a proposition that equal weights of two datasets combined into a dataset have maximum linkage information and minimum error for linkage analysis is given. (DOCX 41 kb) [file 12859_2017_1804_MOESM3_ESM.docx]

**Appendix B**

Let $X=(x_{1},\ldots,x_{n})$be a set of random variables with expectation $\mu$ and variance$\sigma^{2}(X)$, $Y=(y_{1},\ldots,y_{n})$be another set of random variables with expectation $\mu$ and variance$\sigma^{2}(Y)$. Let be a set of new variables with expectation $u=a\mu+b\mu$ and $\sigma^{2}(Z)$where *a* is in [0, 1] and *b* =$1-a$. We then have

$\begin{matrix} \sigma^{2}(Z)=\sum_{i=1}^{n} (Z_{i}-u)^{2}/(n-1)=\sum_{i=1}^{n} [(ax_{i}+by_{i})-(a+b)\mu]^{2}/(n-1) \\ \begin{matrix} & \end{matrix}=\sum_{i=1}^{n} [(ax_{i}-a\mu)+(by_{i}-b\mu)]^{2}/(n-1) \\ \begin{matrix} & & = \end{matrix}a^{2}\sigma^{2}(X)+b^{2}\sigma^{2}(Y)+2ab\sigma^{2}(X,Y) \end{matrix}$ (B1)

where $\sigma^{2}(X,Y)$is covariance of *X* and *Y* and equal to 0 if *X* is independent of *Y*, otherwise, $\sigma^{2}(X,Y)$>0. For *X*=$\hat{Q}_{k}^{*}$and *Y*=$\hat{Q}_{k}$, if $\sigma^{2}(X,Y)$>0, then $\hat{Q}_{k}^{*}$ provides the linkage information for linkage analysis of these three loci. Thus, correlation coefficient $R=\sigma^{2}(X,Y)/\sqrt{\sigma^{2}(X)\sigma^{2}(Y)}$ between *X* and *Y* can be used to measure linkage information between *X* and *Y* for three-point linkage analysis. The simulated results show that the correlation coefficients between $\hat{Q}_{k}^{*}$ and$\hat{Q}_{k}$are 0.966, 0.962, and 0.943 for *k* = 2, 3, 4, respectively, indicating that$\hat{Q}_{k}^{*}$ has almost the same linkage information with $\hat{Q}_{k}$. In addition, $a=b$ leads $2ab\sigma^{2}(X,Y)$to be maximum but $a^{2}\sigma^{2}(X)+b^{2}\sigma^{2}(Y)$ to be minimum for a given$\sigma^{2}(Z)$, i.e., $\hat{Q}_{k}^{*}$ has maximum linkage information and minimum error at *a = b* for linkage analysis.
